# Release kinetics of tumor necrosis factor-α and interleukin-1 receptor antagonist in the equine whole blood

**DOI:** 10.1186/s12917-016-0742-4

**Published:** 2016-06-17

**Authors:** Simon Rütten, Gerald F. Schusser, Getu Abraham, Wieland Schrödl

**Affiliations:** Institute of Pharmacology, Pharmacy and Toxicology, Faculty of Veterinary Medicine, Leipzig University, An den Tierkliniken 15, 04103 Leipzig, Germany; Department of Large Animal Medicine, Faculty of Veterinary Medicine, Leipzig University, An den Tierkliniken 11, 04103 Leipzig, Germany; Institute of Bacteriology and Mycology, Faculty of Veterinary Medicine, Leipzig University, An den Tierkliniken 29, 04103 Leipzig, Germany

**Keywords:** Horse, TNF-α, IL-1receptor antagonist, ELISA, Whole blood culture, Inflammation

## Abstract

**Background:**

Horses are much predisposed and susceptible to excessive and acute inflammatory responses that cause the recruitment and stimulation of polymorphnuclear granulocytes (PMN) together with peripheral blood mononuclear cells (PBMC) and the release of cytokines. The aim of the study is to develop easy, quick, cheap and reproducible methods for measuring tumor necrosis factor alpha (TNF-α) and interleukin-1 receptor antagonist (IL-1Ra) in the equine whole blood cultures ex-vivo time- and concentration-dependently.

**Results:**

Horse whole blood diluted to 10, 20 and 50 % was stimulated with lipopolysaccharide (LPS), PCPwL (a combination of phytohemagglutinin E, concanavalin A and pokeweed mitogen) or equine recombinant TNF-α (erTNF-α). TNF-α and IL-1Ra were analyzed in culture supernatants, which were collected at different time points using specific enzyme-linked immunosorbent assays (ELISA). Both cytokines could be detected optimal in stimulated 20 % whole blood cultures. TNF-α and IL-1Ra releases were time-dependent but the kinetic was different between them. PCPwL-induced TNF-α and IL-1Ra release was enhanced continuously over 24–48 h, respectively. Similarly, LPS-stimulated TNF-α was at maximum at time points between 8–12 h and started to decrease thereafter, whereas IL-1Ra peaked later between 12–24 h and rather continued to accumulate over 48 h. The equine recombinant TNF-α could induce also the IL-1Ra release.

**Conclusions:**

Our results demonstrate that similar to PCPwL, LPS stimulated TNF-α and IL-1Ra production time-dependently in whole blood cultures, suggesting the suitability of whole blood cultures to assess the release of a variety of cytokines in health and diseases of horse.

## Background

Tumor necrosis factor α (TNF-α), in addition to being cytotoxic for certain tumor cells [1, 2], has turned out to be a pro-inflammatory cytokine that is involved in the regulation of immunity and several inflammatory diseases in humans [3, 4] and animals including horses [5, 6]. These animals are much predisposed and susceptible to excessive and acute inflammatory responses that cause the recruitment and stimulation of polymorphnuclear granulocytes (PMN) and peripheral blood mononuclear cells (PBMC) as frequently observed in gastrointestinal diseases [7], laminitis [8], recurrent airway obstruction [9, 10] and endometritis [11]. In horses as well as in humans, the systemic release of TNF-α appears to be an essential early mediator in inflammatory and immunologic reactions during host defense [12–14]. Also, several studies have shown a direct correlation between disease severity/lethality and TNF-α concentration [7, 15, 16].

Moreover, it has been described that TNF-α can orchestrate the production of other cytokines such as interleukin (IL)-1, IL-6 and IL-8 to promote inflammation including increased leukocyte extravasation [17, 18], cellular and tissue damage in multiple organs and clinical features of sepsis [19, 20], both in experimental animal disease models and in human diseases. Evidence exists that excessive production of the cytokines TNF-α and IL-1 significantly contributes to the development of multiple organ damage in endotoxemia and sepsis in humans [21], in the equine endometritis [22], equine asthma [5, 23] and colic [7].

The elevated TNF-α and IL-1 levels trigger often high production of the interleukin-1 receptor antagonist (IL-1Ra), the first described naturally occurring cytokine or hormone-like molecule that functions as a specific IL-1 receptor antagonist and produced by various cell types, including mononuclear cells and neutrophils [24, 25]. IL-1Ra is elevated in plasma of human patients with a variety of inflammatory, infectious, and post-surgical conditions, indicating the importance of hepatic production of this anti-inflammatory protein (for review see reference [26]). Also, administered LPS increased the plasma concentration of IL-1 and concomitantly of IL-1Ra [27], suggesting highly likely that IL-1Ra diffuses from the circulation into tissues and influences the local ratio of IL-1Ra to IL-1 to suppress competitively the IL-1, subsequently to attenuate IL-1-induced TNF-α and IL-6 production [25, 28]. In vitro, it has been also shown that TNF is capable of inducing the production of IL-1Ra [29, 30]. TNF-α has been described to be associated with the human endotoxemia and several inflammatory disorders, and blocking of this cytokine seems to inhibit endotoxin-induced IL-1Ra release [21]; indeed, this relationship to TNF-α has not been examined in horses.

Generally, in the horse, cytokine production can be influenced by several factors including various nutritional, physiological and pathological factors as well as drugs; thus, there is still much interest in measuring the concentrations of different cytokines. In man, successful ex-vivo ELISA-based method of TNF-α measurement has been well established in whole blood system [17, 31] which is quick, less expensive than the analysis in isolated cell systems and can be adapted to field use. In contrast, in horses, current literature review indicates that different matrices have been used to assess TNF-α production ex vivo which did not warrant the generation of uniform data; for example, in bronchoalveolar lavage fluid (BALF) [32], serum [15], plasma [33] or isolated and cultured leukocytes [34–36]. Indeed, different protocols showed culturing PMN influences cell functions and presumably cytokine release [37]. Despite the fact that there are also some studies that used equine whole blood to assess cytokine releases, little was attempted to assess systematically the kinetics of TNF-α production in relation to IL-1Ra production and in different volumes of the whole blood as specialized circulating connective tissue by retaining all blood components.

Thus, we have used the equine whole blood cultures to investigate: first, the relationship between cultures of equine whole blood dilutions and stimulated TNF-α and IL-1Ra release; second, to characterize time-concentration-responses of TNF-α and IL-1Ra production within individuals and different blood dilutions; third, effects of lipopolysaccharide (LPS), a combination of phytohemagglutinin E, concanavalin A and pokeweed mitogen (PCPwL) and equine recombinant TNF-α (erTNF-α) on TNF-α and IL-1Ra in whole blood cultures. Finally, we wished to evaluate, to our knowledge the first time, whether elevated TNF-α can enhance the IL-Ra activity in the equine whole blood cultures. Our results indicate that TNF is involved not only in the secretion of agonistic members of the cytokine network in endotoxemia but also in that of an important antagonistic member (i.e., IL-1Ra), assuming that blockade of TNF-α results in a general down-regulation of the cytokine system.

## Methods

### Animals

For the kinetic time-course studies of TNF-α and IL-1Ra releases, blood samples were collected from three-five healthy horses of mixed breeds and 8–14 years of age. They were neither under treatment for the last three weeks before sampling nor were given immune-suppressing drugs. Animals were kept in an individual pen, fed with hey and concentrates three times a day, but had access to water ad libitum. Blood samples were collected from horses, which belong to the Large Animal Clinic of the Leipzig University.

### Reagent and antibody dilutions

TNF-α antibodies^1^ were reconstituted to final stock concentrations of 1 mg/ml (capture) and 0.25 mg/ml (detection) in ultra-pure water.^2^ Complete RPMI 1640 medium contained basal RPMI 1640 medium^2^, Penicillin G^2^ (100 U/ml), Streptomycin^2^ (100 μg/ml) and heparin^2^ (10 U/ml). The TNF-α-capture antibody was diluted in 0.1 M NaHCO_3_ solution with ultra-pure water to a working concentration of 3 μg/ml. The detection antibody was diluted to a working concentration of 250 ng/ml in PBST-buffer, containing 2 % heat inactivated (56 °C, 30 min) and sterile-filtered rabbit serum.^3^ Streptavidin-horseradish-peroxidase^4^ (HRP) was diluted 1:50000 in PBST containing Dulbecco’s phosphate buffered saline (PBS) solution^5^ and 0.1 % Tween 20.^6^ With regard to IL-1Ra, antibodies and reagents were processed in accordance with the manufacturer’s instructions (Equine IL-1ra/IL-1 F3 DuoSet ELISA).^7^

### Blood sampling, whole blood cell culture and stimulation

Blood samples were collected via jugular venipuncture into 9 mL vacutainer tubes with Li-Heparin (16 U × mL^−1^) (S-Monovette).^8^ Blood dilutions, culturing and whole blood stimulations were carried out under sterile conditions. Within 30 min after sampling, whole blood was diluted at a ratio of 1:2 (50 %), 1:5 (20 %) and 1:10 (10 %) in a complete RPMI 1640 medium, respectively.

For the stimulation of TNF-α and IL-1Ra releases, diluted blood was incubated in 24-well culture plates (total volume 2 ml/well). Samples were stimulated with either LPS^9^ (1000 ng/ml; Escherichia coli O111:B4) or with PCPwL (100 ng/ml) (a combination of phytohemagglutinin E,^10^ concanavalin A^10^ and pokeweed mitogen^10^) (as positive control) or with medium alone. Moreover, whole blood cultures were treated with the equine recombinant TNF-α^11^ (erTNF-α; 20 ng/ml) to stimulate IL-1Ra antagonist release. Each culture plate was then incubated for 72 h at 37 °C under 5 % CO_2_ atmosphere. To assess the time course of cytokine releases, aliquots of the supernatant/well were collected at time points of 0, 1, 2, 4, 6, 8, 12, 16, 24, 48 and 72 h. These aliquots were centrifuged (10000 rpm) at 4 °C for 3 min and the supernatant was separated from the cell debris and frozen at −20 °C until analysis of the cell-associated cytokines.

### Detection of TNF-α and IL-1Ra production

Concentrations of cytokines in whole blood cell culture supernatants were determined by enzyme-linked immunosorbent assay (ELISA) system. All samples from each blood dilutions were analyzed in one ELISA.

#### Measurement of TNF-α

The TNF-α protein level was measured using the equine TNF-α sandwich ELISA that was established in our laboratory. For this purpose, 96-microtiter well plates^12^ were coated with 100 μl/well of a polyclonal antibody specific for equine TNF-α and incubated overnight at 4–6 °C. Thereafter, samples were allowed to reach room temperature. Plates were then washed twice with 400 μl/well washing buffer (distilled water containing 0.9 % NaCl and 0.05 % Tween 20) using an automated 8-channel washer.^13^

Assay conditions were optimized by adding a series of calibrators on every assay plate that consisted of 7-point serial dilutions of equine recombinant TNF-α starting from 24.3 ng/ml to 0.033 ng/ml in duplicates in complete RPMI 1640 cell culture medium containing PBST in the presence of 10, 20 or 50 % horse serum. Also, duplicate wells of each plate contained only medium. For each ELISA plate, the optical density (OD) at 450 nm was measured, and the standard curves were established by plotting mean absorbance for each standard concentration against the target protein concentration with OD 450 nm as the Y-axis using GraphPad Prism Software program. These curves were used to determine protein expression levels.

Basal and stimulated levels of TNF-α and IL-1Ra in whole blood culture supernatant samples were measured using ELISA. In brief, for each point of assay, 50 μl of pure cell culture supernatant were pipetted into each well of 96-microtiter well plates and diluted 1:2 by adding 50 μl PBST-buffer. Samples were then incubated for 1 h at room temperature. After three times rinsing the plates with washing buffer, 100 μl of the polyclonal biotin-labeled detecting antibody were added to each sample and incubated for further 2 h at room temperature. After three washing steps, plates were incubated with 100 μl horseradish peroxidase-conjugated streptavidin for 1 h at room temperature. After four washing steps, 100 μl of freshly prepared substrate solution (containing 3 mM H_2_0_2_ and 1 mM tetramethylbenzidine^14^ (TMB) in 0.2 M citrate buffer, pH 4.0) were added to each well. The chromogenic substrate was converted from colorless to blue by streptavidin-HRP conjugate. The reaction was stopped after 15 min at room temperature by adding 50 μl of 1 M sulfuric acid solution, and the optical density (OD) was read at 450 and 620 nm with a spectrophotometer (Tecan Plate reader^15^). The levels of TNF-α in whole blood supernatant samples were interpolated from the erTNF-α standard calibration curves calculated as described above.

#### Measurement of IL-1Ra

IL-1Ra concentrations were measured in whole blood culture supernatants according to the manufacturers’ guidelines with slight modifications. Briefly, 96-microtiter well plates were coated with polyclonal equine anti-IL-1Ra antibody and incubated overnight at room temperature, followed by washing and blocking steps. Freshly thawed whole blood supernatant samples and the equine IL-1Ra standard (at different concentrations starting from 40 ng/ml to 0.16 ng/ml) were diluted at a ratio of 1:10 and incubated. Following several washing steps, biotinylated secondary IL-1Ra antibody, streptavidin-HRP conjugate and enzyme substrate were added at different time points as given in the guidelines. Further procedures were performed analogous to the TNF-α ELISA.

### Data calculation and statistical analysis

Results are presented as means ± SEM. The levels of TNF-α and IL-1Ra in whole blood supernatants were interpolated from the erTNF-α and IL-1Ra standard calibration curves which were established using GraphPad Prism 6.01^16^. Variations in cytokine productions in the different whole blood cultures and time courses of incubation as well as different cytokine stimulants were assessed by two-way analysis of variance (ANOVA) using SigmaPlot 12.5^17^ with Holm-Sidak post-hoc test. Cytokine productions in different culture conditions were plotted and statistical significance was calculated. Differences giving *P* value < 0.05 were considered significant.

## Results

### Relationship between whole blood dilutions and cytokine releases

The ex vivo production of TNF-α and IL-1Ra was determined in horses using heparin-displaced whole blood samples collected in a single draw. Samples were diluted 1:2 (50 %), 1:5 (20 %) and 1:10 (10 %) with complete RPMI 1640 medium. The PCPwL- but also LPS-stimulated TNF-α and IL-1Ra production was linear with different concentrations of the equine whole blood, whereby the highest cytokine concentration was achieved at whole blood dilution of 50 %. In all whole blood dilutions, PCPwL stimulated continuously TNF-α release over 24 h to the maximum and similarly but within 12 h of stimulation, LPS activated TNF-α production (Table 1). The stimulation with these both stimulating agents and additionally with erTNF-α yielded on average a maximum IL-1Ra production in all whole blood dilutions over 48 h of stimulation (Table 2). Despite the linearity of ELISA signals for TNF-α and IL-1Ra as a function of whole blood dilution, there were high background signals and strong adherence of diluted blood in the pipette tips at a volume of 50 % (with large variation in cytokine concentration). These errors were considered significant; thus, in all subsequent experiments, 20 % equine whole blood dilution, which provided low data variation of cytokine concentration, was routinely used, since also 10 % dilution tended not to result in reliable data.

**Table 1:** Maximal TNF-α concentration after stimulation with LPS or PCPwL

BC (%)	LPS	PCPwL	
	8 h	24 h	72 h
10 %	296 ± 153	103 ± 103	345 ± 106
20 %	538 ± 219	304 ± 164	517 ± 163
50 %	1104 ± 279	865 ± 187	661 ± 316

Supernatant TNF-α level (pg/ml) was compared between different whole blood volumes after stimulation with LPS or PCPwL. Maximal concentrations were obtained after the start of stimulation at various time points until 72 h. Data represent means ± SEM of three different horses. BC, blood concentration

**Table 2:** Maximal IL-1Ra production after stimulation with LPS, PCPwL or erTNF- α

BC (%)	LPS		PCPwL	erTNF-α	
	48 h	72 h	48 h	48 h	72 h
10 %	5643 ± 1520	5809 ± 1426	8045 ± 2965	5083 ± 2364	5120 ± 2323
20 %	11368 ± 1713	8502 ± 1667	11451 ± 1620	11167 ± 994	8829 ± 178
50 %	26375 ± 4842	21260 ± 7332	25982	>40000	27529 ± 4010

Supernatant IL-1Ra production (pg/ml) was compared between different whole blood volumes after stimulation with LPS, PCPwL or erTNF-α. Maximal concentrations were obtained after the start of stimulation at various time points until 72 h. Data represent means ± SEM of three different horses. BC, blood concentration

### Time-course kinetics of TNF-α release in whole blood cell culture

**Fig. 1:**
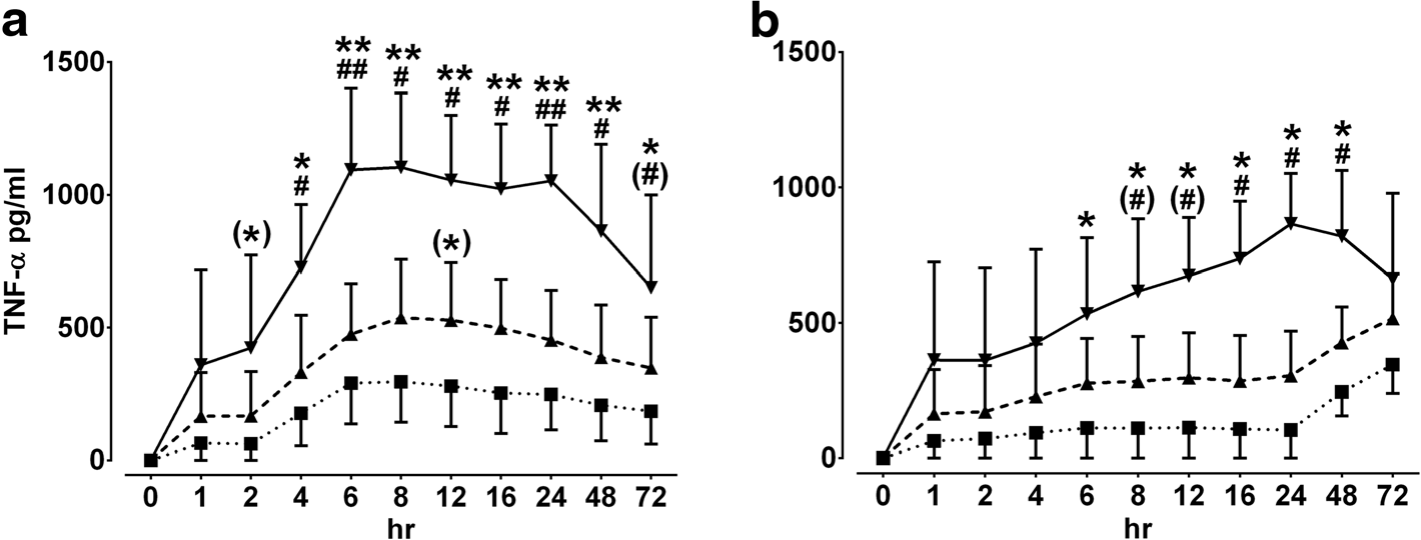
Effect of time and blood dilution on TNF-α production in whole blood culture. Time-dependent TNF-α production was measured in equine whole blood cell culture supernatants obtained from different blood volume dilutions after stimulation by LPS (1000 ng/ml) (**a**) and PCPwL (100 ng/ml) (**b**). 10 % (■), 20 % (▲), and 50 % (▼) whole blood volumes were used to assess TNF-α over 72 h (hr). Data represent means ± SEM of three different horses. ** *p* < 0.001/* *p* < 0.05/(*) 0.05 ≥ *p* < 0.1 versus 10 % BC, ^##^ *p* < 0.001/^#^ *p* < 0.05/(^#^) 0.05 ≥ *p* < 0.1 versus 20 % BC. BC, blood concentration

**Fig. 2:**
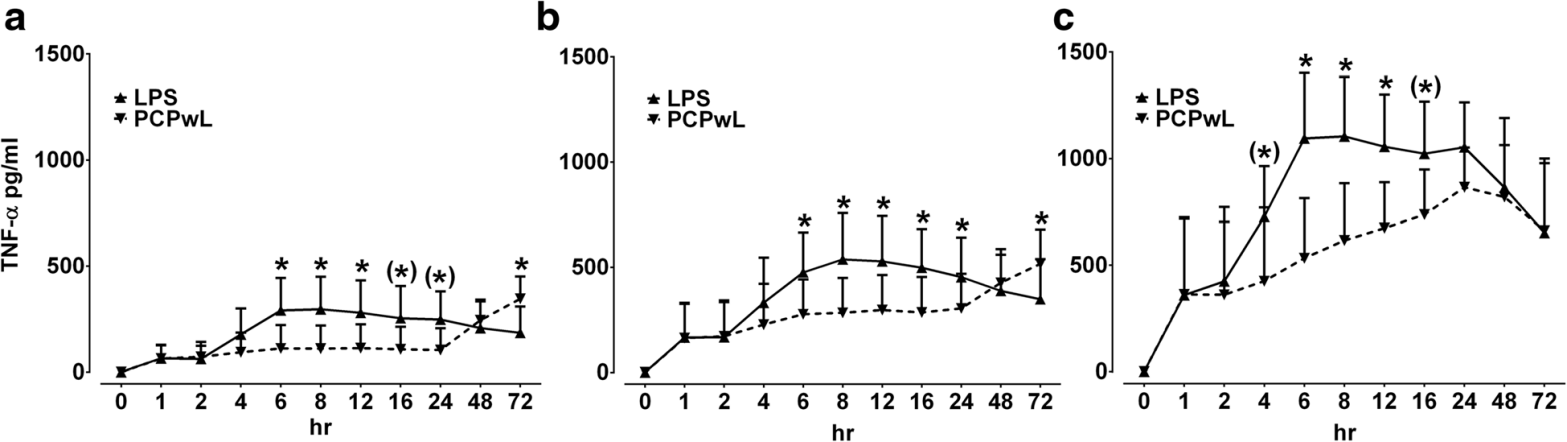
Comparison of LPS- and PCPwL-stimulated TNF-α production. LPS- and PCPwL-stimulated TNF-α production was measured in 10 % (**a**), 20 % (**b**) and 50 % (**c**) whole blood cultures over time. Data represent means ± SEM of three different horses. ** *p* < 0.001/* *p* < 0.05/(*) 0.05 ≥ *p* < 0.1. BC, blood concentration

In the next step, the relationship between equine whole blood dilutions and time-course of LPS-stimulated TNF-α production in comparison to PCPwL was examined. As depicted in Figs. 1 and 2, the mean production of TNF-α in relation to whole blood dilution was affected by time. One hour after LPS addition, the level of TNF-α started to increase and maximum levels were achieved at the incubation time between 8–12 h. Thereafter, TNF-α concentration started to decrease continuously up to 72 h (Fig. 1a). In whole blood cultures of both 10 and 20 % dilutions, PCPwL stimulated the TNF-α production to a similar extent as LPS, but the concentration of TNF-α increased gradually and peaked until up to 72 h while in 50 % diluted whole blood cultures TNF-α level declined continuously after 24 h of incubation (Fig. 1b). Comparing both stimulating agents, LPS seems to stimulate TNF-α production more than PCPwL, and this was also dependent on whole blood dilutions (Fig. 2). Together, due to the data fluctuation of TNF-α releases, which were obtained at whole blood dilutions of 10 or 50 % and the variable peak time courses obtained after PCPwL and also after LPS stimulation, 12-h incubation is assumed to be an optimal condition to measure TNF-α in 20 % whole blood cultures.

### Time-course kinetics of IL-1Ra activation

**Fig. 3:**
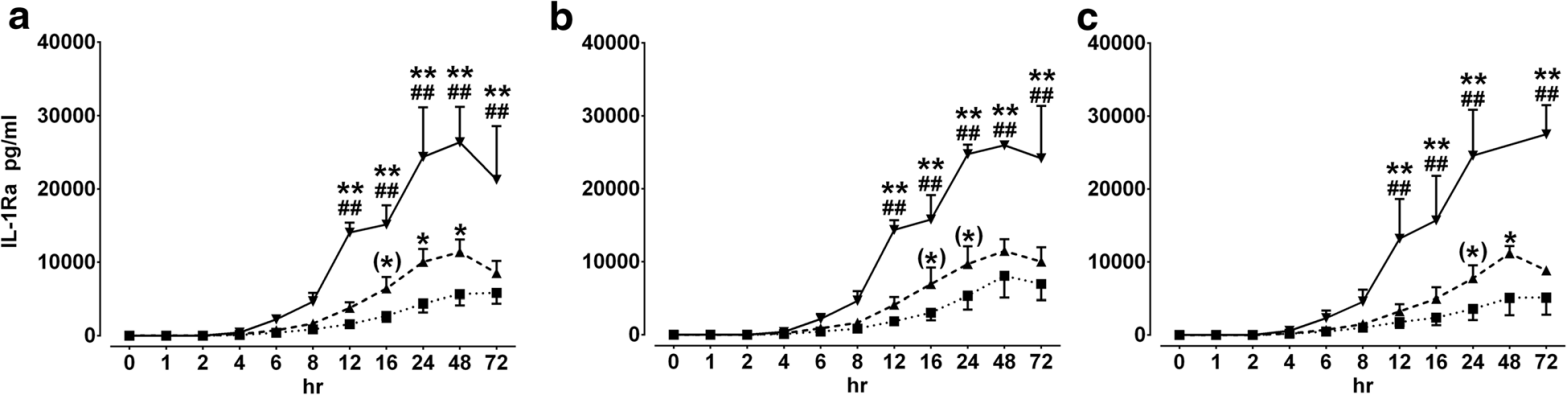
Effect of Time and dilution on IL-1Ra production in whole blood culture. IL-1Ra production was measured in equine whole blood cell culture supernatants obtained from different blood volume dilutions (10 % (■), 20 % (▲), and 50 % (▼)) stimulated by LPS (1000 ng/ml) (**a**) PCPwL (100 ng/ml) (**b**) and erTNF-α (20 ng/ml) (**c**) over 72 h. Data represent means ± SEM of three different horses. over 72 h (hr). ** *p* < 0.001/*p < 0.05/(*) 0.05 ≥ *p* < 0.1 versus to 10 % BC, ^##^ *p* < 0.001/^#^ *p* < 0.05/(^#^) 0.05 ≥ *p* < 0.1versus to 20 % BC; BC, blood concentration

**Fig. 4:**
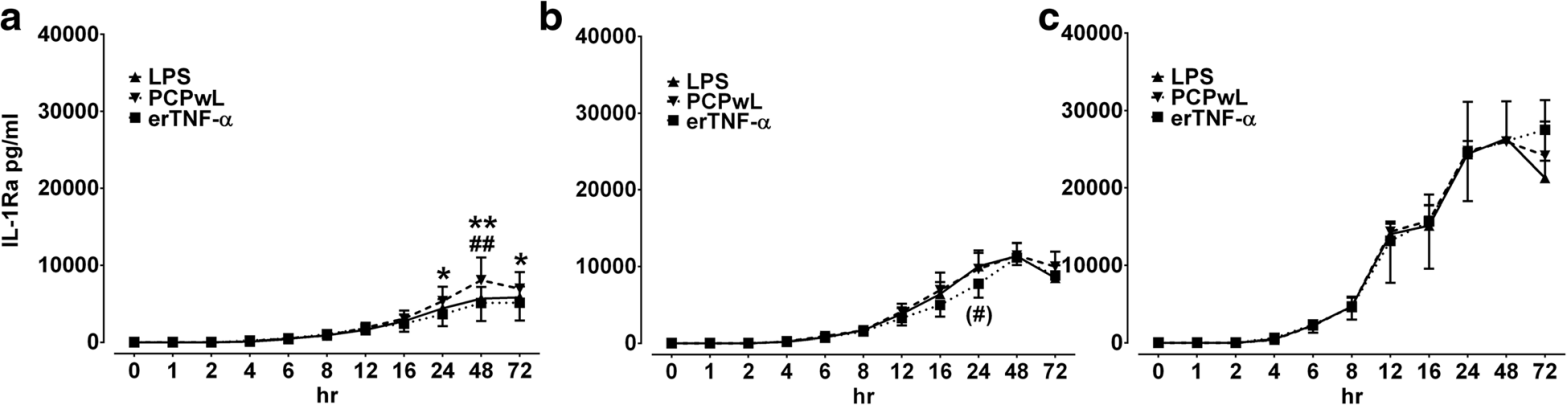
Comparison of LPS-, PCPwL- and erTNF-α-stimulated IL-1Ra production. LPS-, PCPwL- and erTNF-α-stimulated IL-1Ra production was measured in 10 % (**a**), 20 % (**b**) and 50 % (**c**) whole blood cultures over time. Data represent means ± SEM of three different horses. **p < 0.001/*p < 0.05/(*) 0.05 ≥ *p* < 0.1. BC, blood concentration

Strong and significant activation of IL-1Ra release was obtained in 50 % diluted equine whole blood cultures regardless of the used stimulating agents (LPS, PCPwL or erTNF-α) (Fig. 3a-c). Interestingly, it was able to detect IL-1Ra release first after about 4 h of stimulation of the whole blood cultures of 20 and 50 % dilutions and the maximum response was obtained after 48 h and declined after 72 h. Similar data of IL-1Ra release were obtained when 10 % whole blood cultures were stimulated but maximum response was obtained here after about 72 h of incubation. As shown in Fig. 4, there were little significant differences between IL-1Ra levels obtained from three whole blood dilutions stimulated by all three stimulating agents (LPS, PCPwL or erTNF-α) over 72 h.

## Discussion

The present study dissects equine whole blood dilution-dependent and time-course secretion of TNF-α and IL-1Ra after stimulation by PCPwL, LPS as well as erTNF-α (only IL-1Ra was stimulated) ex vivo. The decisive results were: PCPwL and also LPS triggered TNF-α and IL-1Ra releases in a blood volume-dependent manner (50 % > 20 % >10 %). In whole blood cultures, TNF-α exhibited a kinetic profile similar to that of IL-1Ra, but peaked explicitly prior to IL-1Ra activity. TNF-α peaked 8–12 h after LPS addition while the IL-1Ra level was at maximum 24 h later and remained at that level for further 24 h. The effect of several stimuli on cytokine releases was often investigated in isolated leukocytes of horses [34–36] and humans [38, 39]. Also, cytokines could be assessed in human whole blood cultures [31, 37–39], but to our knowledge, very rarely or even not analyzed the time-dependent kinetics of TNF-α release in relation to IL-1Ra production in an equine whole blood-volume-dependent manner.

For reasons that the optimal stimulation conditions to assess cytokine releases in the equine whole blood cultures were not well known, the aim of the current study was to first find out which blood volumes can provide a reproducible signal-to-noise ratio of cytokine standard and sample concentration curves. In the whole blood medium, which resembles closely the physiological milieu, it was observed that the PCPwL- or LPS-stimulated TNF-α and IL-1Ra release was reduced in whole blood cultures with increasing dilutions (cf. Tables 1 and 2). Indeed, the data obtained from whole blood cultures of 1:2-diltution (50 %-vol.) were subjected fluctuations, presumably due to more background signals as a result of strong adherence of the blood to the pipette tips when compared to the data obtained from equine whole blood cultures of the 20 and 10 % dilutions. Even if 10 % whole blood samples would require smaller starting blood samples to assess cytokines, signals obtained here were weak. On the other hand, the 1:5-dilution (20 %-vol.) of the whole blood delivered consistently high reproducible cytokine data. This validated novel, cheap, optimized and straightforward approach was further used to quantify TNF-α and IL-1Ra in whole blood cultures of the horse, and can be suggested for the use in field conditions. Our data are in agreement with data from earlier studies that demonstrated stable cytokine production in diluted human whole blood cultures (dilution ranged: 1:4 to 1:10) [31, 38, 39]. These authors compared whole blood cultures with isolated monocytes or PBMC and showed constant cytokine reproducibility in whole blood cultures than in isolated cells, thus, suggested that whole blood is a better suited medium than isolated blood cell cultures for cytokine studies in humans. Even though in the current study leukocytes were not isolated, it can be suggested that large-scale ex vivo cytokine assessment can be undertaken with equine whole blood cultures.

Similar to the effect of whole blood dilution on equine TNF-α and IL-1Ra production, there was an effect of time on the release of both cytokines ex vivo after stimulation with PCPwL or LPS. With regard to TNF-α, the release kinetics in whole blood stimulation ex vivo were similar to those reported in vivo after LPS-challenge of horses [40–42]. In these in vivo models of endotoxemia, the low-dose LPS challenge of horses resulted in a predictable effect on increasing plasma TNF-α activity that peaked between 1–4 h (average 1.5–2.5 h). In our study, cytokine levels increased after 1-h stimulation of the equine whole blood cultures with LPS and peaked between 8–12 h. This time was considered as an optimal incubation condition for further experiments, since stimulation of blood cultures for longer times resulted in decreased TNF-α production e.g. until 72 h.

Interestingly, we could also demonstrate the first time that IL-1Ra can be detected in relation to TNF-α production in the equine whole blood cultures and can be also affected with time. Both PCPwL and LPS could induce a continuous production of IL-1Ra over 48 h, indicating that the elevated TNF-α may contribute to the high level of IL-1Ra even beyond the peak time points, which were observed for the former cytokine. The activated IL-1Ra levels were much higher than the levels of TNF-α, presumably, to block the biological effects of IL-1 [25, 27] which is concomitantly released during inflammatory processes in several tissue and organ systems. Furthermore, in the current study, stimulation of the equine whole blood cultures with the equine recombinant TNF-α (erTNF-α) resulted in enhanced activity of IL-1Ra, in agreement with the data obtained from human studies [43–45]. In man, this relationship has been shown during endotoxemia [21] and high level of acute-phase protein [46, 47] and could be linked to the involvement of TNF-α in the course of several diseases. However, in horses, excessive IL-1Ra production in relation to TNF-α release has not yet been associated with disease pathogenesis and severity. In the first place, IL-1Ra is claimed to attenuate the IL-1-dependent activities [25] which are necessary in tissue remodeling which often occur in several fibrotic equine diseases such as in the recurrent airway obstruction (RAO).

## Conclusions

Together, this study indicates the suitability of whole blood cultures to assess ex vivo or in vivo the production of a variety of cytokines of healthy or diseased horses (e.g. inflammatory disorders of the airways, gastro-intestinal tract, uterus, liver etc.), presumably, more than isolated PMN or PBMC cultures. The equine whole blood assay system is additionally an efficient tool to elucidate interactions of cytokines within the inflammatory responses and may provide equine veterinarians with new therapeutic approaches. Dilution of the whole blood to a ratio of 1:5 (20 %) and an incubation time of 12 h can be considered as optimal conditions to assess stimulated cytokines.

## Abbreviations

BALF, Bronchoalveolar lavage fluid; ELISA, Enzyme-linked immunosorbent assay; erTNF-α, Equine recombinant tumor necrosis factor alpha; HRP, Horseradish-peroxidase; IL, Interleukin; IL-1Ra, Interleukin-1 receptor antagonist; LPS, Lipopolysaccharide; PBMC, Peripheral blood mononuclear cells; PBST, Solution containing phosphate buffered saline and Tween 20; PCPwL, Combination of phytohemagglutinin E, concanavalin A and pokeweed mitogen; PMN, Polymorphnuclear granulocytes; TMB, Tetramethylbenzidine; TNF-α, Tumor necrosis factor alpha

## Footnotes

1 ThermoFisher Scientific, Rockford, Illinois, USA

2 Biochrom, Berlin, Germany

3 Biowest, Nuaille’, France

4 ThermoFisher Scientific, Rockford, Illinois, USA

5 Biochrom, Berlin, Germany

6 Roth, Karlsruhe, Germany

7 R&D Systems, Wiesbaden, Germany

8 Sarstedt, Nümbrecht, Germany

9 Sigma-Aldrich, Taufkirchen, Germany

10 Biochrom, Berlin, Germany

11 Perbio science, Rockford, Illinois, USA

12 BD Bioscience, Heidelberg, Germany

13 Nunc, Roskilde, Denmark

14 Sigma-Aldrich, Taufkirchen, Germany

15 Tecan, Crailsheim, Germany

16 Graphpad Software Inc., La Jolla, California, USA

17 Systat Software Inc., San Jose, California, USA
